# A human Dravet syndrome model from patient induced pluripotent stem cells

**DOI:** 10.1186/1756-6606-6-19

**Published:** 2013-05-02

**Authors:** Norimichi Higurashi, Taku Uchida, Christoph Lossin, Yoshio Misumi, Yohei Okada, Wado Akamatsu, Yoichi Imaizumi, Bo Zhang, Kazuki Nabeshima, Masayuki X Mori, Shutaro Katsurabayashi, Yukiyoshi Shirasaka, Hideyuki Okano, Shinichi Hirose

**Affiliations:** 1 Department of Pediatrics, School of Medicine, Fukuoka University, 45-1, 7-chome, Nanakuma, Jonan-ku, Fukuoka, 814-0180, Japan; 2 Central Research Institute for the Pathomechanisms of Epilepsy, Fukuoka University, Fukuoka, Japan; 3 Department of Pediatrics, Jikei University Scool of Medicine, Tokyo, Japan; 4 Department of Neurology, University of California, Sacramento, CA, USA; 5 Department of Cell Biology, Fukuoka University School of Medicine, Fukuoka, Japan; 6 Department of Physiology, Keio University School of Medicine, 35 Shinanomachi, Shinjuku-ku, Tokyo, 160-8582, Japan; 7 Kanrinmaru-Project, Keio University School of Medicine, 35 Shinanomachi, Shinjuku-ku, Tokyo, 160-8582, Japan; 8 Department of Biochemistry, Fukuoka University School of Medicine, Fukuoka, Japan; 9 Department of Pathology, Fukuoka University School of Medicine, Fukuoka, Japan; 10 Department of Physiology, Fukuoka University School of Medicine, Fukuoka, Japan; 11 Department of Neuropharmacology, Faculty of Pharmaceutical Sciences, Fukuoka University, Fukuoka, Japan; 12 Shirasaka Clinic, Kobe, Japan

**Keywords:** Induced pluripotent stem cells, Disease modeling, Dravet syndrome, *SCN1A*, Nav1.1, Epileptogenesis, Action potential, Gamma aminobutyric acid

## Abstract

**Background:**

Dravet syndrome is a devastating infantile-onset epilepsy syndrome with cognitive deficits and autistic traits caused by genetic alterations in *SCN1A* gene encoding the α-subunit of the voltage-gated sodium channel Na_v_1.1. Disease modeling using patient-derived induced pluripotent stem cells (iPSCs) can be a powerful tool to reproduce this syndrome’s human pathology. However, no such effort has been reported to date. We here report a cellular model for DS that utilizes patient-derived iPSCs.

**Results:**

We generated iPSCs from a Dravet syndrome patient with a c.4933C>T substitution in *SCN1A*, which is predicted to result in truncation in the fourth homologous domain of the protein (p.R1645*). Neurons derived from these iPSCs were primarily GABAergic (>50%), although glutamatergic neurons were observed as a minor population (<1%). Current-clamp analyses revealed significant impairment in action potential generation when strong depolarizing currents were injected.

**Conclusions:**

Our results indicate a functional decline in Dravet neurons, especially in the GABAergic subtype, which supports previous findings in murine disease models, where loss-of-function in GABAergic inhibition appears to be a main driver in epileptogenesis. Our data indicate that patient-derived iPSCs may serve as a new and powerful research platform for genetic disorders, including the epilepsies.

## Background

Dravet syndrome (DS) is an infantile-onset epileptic encephalopathy that develops in a previously normal infant [1]. Seizures are refractory to all currently available forms of treatment; severe neuropsychiatric disabilities include cognitive deficits and autism-spectrum behaviors, and approximately 10–20% of the afflicted children do not survive [2,3]. Clearly, new and improved treatment modalities are needed, but their development hinges on research platforms that faithfully reproduce the human pathology.

Defects in the *SCN1A* gene, which encodes the α-subunit of the voltage-gated sodium channel Na_v_1.1, are seen in 70–80% of patients with DS, and approximately 50% of these defects truncate the Na_v_1.1 protein prematurely [4,5]. Various approaches have been used to describe and characterize the condition, most notably heterologous expression of Na_v_1.1 mutants [6,7] and, more recently, the development of DS mouse models, which are based on heterozygotes of an *Scn1a* knock-out/knock-in [8,9], or cell-type specific conditional knock-out [10,11]. These efforts have revealed the pathogenic mechanism for DS likely involves Na_v_1.1 haploinsufficiency [11-14]. Additionally, in the rodent forebrain, Na_v_1.1 is predominantly expressed in GABAergic interneurons [15], especially in the axon initial segment of a parvalbumin (PV)-positive subgroup [9], where Na_v_1.1 has been suggested to directly influence action potential generation and thereby exert excitation control over downstream pyramidal neurons [16]. Impaired inhibition through disruption of this suppression by forebrain GABAergic neurons may be the main pathogenic mechanism underlying the seizure susceptibility of DS [8-10,17]. A recent study has indicated that autism-related behaviors in *Scn1a*^+/-^ mice result from the impaired GABAergic neurotransmission [11]. The full spectrum of factors contributing to the phenotype, however, is likely more complex with additional, so far unidentified components modifying the presentation.

Various neurological disease models have been developed using patient-derived iPSCs [18-23], but to date, no such effort has been reported toward epilepsy. Due to the early onset of the disorder and the wealth of knowledge on the associated genetic defects, DS is a highly suitable candidate for iPSC technology. We here report the first successful development of an iPSC-based DS model incorporating a nonsense mutation in *SCN1A*, and we show how neurons of this model have abnormal electrophysiological responses.

## Results

### Patient demographics and history

**Figure 1:**
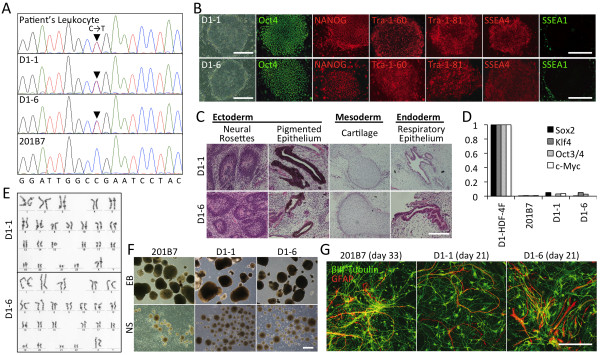
**Characterization of generated iPSCs and neuronal differentiation.** (**A**) *SCN1A* sequencing of the indicated cell material. Solid arrowheads point to the c.4933C>T substitution. (**B**) iPSC morphology and immunostaining of pluripotency markers (Oct 4, Nanog, Tra-1-60, Tra-1-81, and SSEA4 without SSEA1). Scale bar, 500 μm. (**C**) iPSC-derived teratomas generated in NOD-SCID mouse testes comprised tissues from all three germ layers. Scale bar, 200 μm (neural rosettes and respiratory epithelium) and 400 μm (others). (**D**) Real-time PCR analysis showed suppressed expression of the four reprogramming factors in both patient and control iPSCs compared to patient fibroblasts transduced with the same four factors (D1-HDF-4F). (**E**) G-band karyotyping showed normal chromosome numbers (46,XX) in all tested colonies (*N* = 20 each). (**F**) Representative images of embryoid bodies (EB) and neurospheres (NS). Scale bar, 500 μm. (**G**) Expression of βIII-tubulin, a neuronal marker (green) and GFAP, an astrocyte marker (red) in iPSC-derived neural cells. Scale bar, 200 μm. Day numbers indicate the days of differentiation in adherent culture after neurosphere formation.

The tissue donor was a female patient who was born in an uneventful delivery of dizygotic twins at 38 weeks of gestation. Her birth weight was 2850 g. There were no complications during the perinatal and early postnatal period. However, at 6 months of age, she experienced her first seizure with loss of consciousness. At 7 months of age, generalized tonic-clonic seizures began, often prolonged, and induced by fever or hot baths. Despite anticonvulsant therapy, she later developed facial myoclonia and focal seizures, and obtundation status [24]. Generalized polyspikes-waves were identified in interictal electroencephalography. At the age of 21, direct sequencing of blood leukocyte-extracted genomic DNA revealed an *SCN1A* point mutation, c.4933C>T [GenBank: NM_001165963.1] (Figure 1A) as first reported by Fukuma and co-workers [25], which is expected to prematurely truncate the Na_v_1.1 protein in the fourth homologous domain (p.R1645*, Additional file 1) [GenPept: NP_001159435.1]. By 29 years of age, when a skin biopsy was performed, she had developed profound intellectual disability as well as ataxia. At that time, she had 7–8 nocturnal generalized tonic-clonic seizures a month and obtundation status once every 2–3 months.

### Characterization of the generated iPSCs

Two lines of patient-derived iPSCs, D1-1 and D1-6, were established from skin fibroblasts obtained from the biopsy specimen. Control experiments used the iPSC line, 201B7, which was developed from the facial skin of a 36-year-old Caucasian female as reported previously [26]. All iPSC colonies had the typical human embryonic stem cell morphology with tightly-packed cells, a clear border, and a round shape (Figure 1B). Expression of pluripotency markers was confirmed (Figure 1B); additional analysis showed that the resulting teratomas consisted of tridermic tissues (Figure 1C), which supports the iPSCs’ undifferentiated state and pluripotency. Silencing of reprogramming transgenes, normal karyotype (46,XX), and the presence of the *SCN1A* c.4933C> T variation were confirmed (Figure 1D, E, A, respectively). Direct sequencing of additional sodium channels genes, the closely related *SCN2A* as well as the genes for subunits β1 and β2 (*SCN1B* and *SCN2B*) revealed wildtype status for all examined sequence regions (data not shown). During neural induction, all clones efficiently generated neurospheres (Figure 1F). In adherent cells differentiated from neurospheres, the expression of neuron and astrocyte markers was confirmed (βIII-tubulin and GFAP, respectively – Figure 1G). Staining for CNPase, an oligodendrocyte marker was negative in all cell lines.

### Na_v_ expression in iPSC-derived neurons

**Figure 2:**
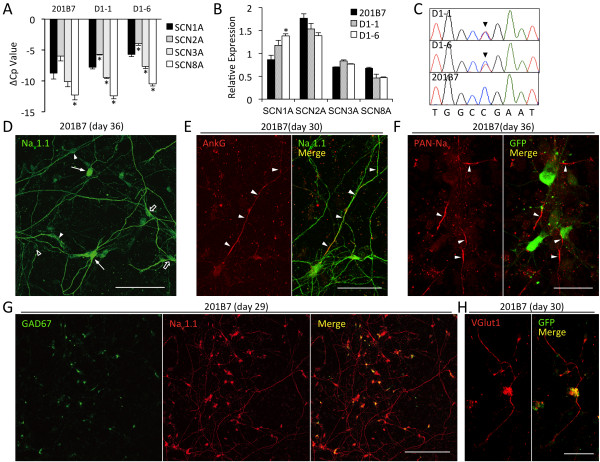
**Na**_**v **_**channel expression in iPSC-derived neurons.** (**A**) Real-time PCR addressing neuronal Na_v_ expression at 30 days of differentiation (*N* = 3 in each cell line) Crossing point differences to β-actin (ΔCp = Cp^*β-actin*^ - Cp^*Nav*^) closer to zero indicate higher expression. PCR efficiencies were nearly identical (Additional file 2). Asterisks indicate a significant difference to *SCN1A* (*P* < 0.5, one-way ANOVA). Expression strength of the indicated Na_v_ genes was constant across the cell lines (*P* = 0.92, two-way ANOVA) (**B**) Normalized expression levels for each Na_v_ gene (*SCN1A* + *SCN2A* + *SCN3A* + *SCN8A*)/4 = 1. Compared to the control, *SCN1A* expression tended to be higher in D1-1 (*P* = 0.0929, one-way ANOVA), and it was significantly higher in D1-6 (^*^P = 0.0078). The distribution of Na_v_ genes expression ratios in each cell line was significantly different between the control and the patient lines (*P* =0.0086 and <0.0001 for D1-1 and D1-6, respectively, two-way ANOVA), but identical between D1-1 and D1-6 (*P* = 0.11). (**C**) Sequencing of *SCN1A* reverse transcribed mRNA isolated from iPSCs-derived neurons. Patient-neurons show a double peak at mutation site (solid arrowheads), confirming the heterozygous state of the cells (**D**) Immunocytochemical characterization of Na_v_1.1 expression in control neurons: strong (solid arrows), moderate (open arrows), weak (solid arrowheads), and faint (open arrowhead). Despite weak staining in the cell body, neurite staining was often apparent (solid arrowheads). (**E**) Neurite co-localization of Na_v_1.1 and the AIS marker ankyrin G (AnkG, solid arrowheads). (**F**) PAN-Na_v_ staining of *SCN1A* Venus-positive neurons (via anti-GFP, see Figure 3) in the AIS (arrowheads). (**G**) Co-localization of Na_v_1.1 and GAD67 staining. (**H**) VGlut1-positive neuron with *SCN1A* Venus expression. Scale bars: 100 μm (**D**), 30 μm (**F**), 200 μm (**G**) and 50 μm (others).

To determine the expression levels for those voltage-gated sodium channels, that predominate in the brain, we used real-time PCR targeting genes *SCN1A*, *SCN2A* (protein name: Na_v_1.2), *SCN3A* (Na_v_1.3), and *SCN8A* (Na_v_1.6) on iPSCs-derived neurons at 30 days of differentiation. In all cell lines, *SCN2A* expression was highest, followed by *SCN1A*, *SCN3A,* and *SCN8A* (Figure 2A and Additional file 2). When the expression levels were normalized to each other, we found that *SCN1A* tended to be expressed higher in the patient neurons than in control neurons (Figure 2B). We furthermore confirmed that, in patient neurons, *SCN1A* mRNA translated from the mutated allele was present (Figure 2C), which suggests that the mutated mRNA was able to escape nonsense-mediated decay, possibly owing to the mutation locating to the last coding exon [27].

We next examined Na_v_1.1 expression at the protein level using a polyclonal antibody targeting the D1-D2 linker (Additional file 1). Among βIII-tubulin-positive cells, Na_v_1.1 immunostaining was identified in 59.0% or 105/178 in 201B7, 52.1% or 139/267 in D1-1, and 58.1% or 151/260 in D1-6 neurons. Na_v_1.1-immunostaining was evident in cell bodies, dendrites, and axons (Figure 2D, E); the antibody’s specificity was confirmed with epitope peptide pre-treatment (Additional file 3). Neurons with well-developed axons often displayed strong axonal Na_v_1.1-staining (Figure 2E). Intense expression of Na_v_ channels (PAN-Na_v_) in the axon initial segment became evident after several weeks of *in vitro* differentiation of neurospheres (Figure 2F, Additional file 4A). This spatial and temporal expression pattern has been suggested to be critical in action potential generation [28].

### Subtypes of Na_v_1.1-positive neurons

The majority of the Na_v_1.1-positive control and patient-derived neurons were GABAergic in nature as established by GAD67 staining (58.3% or 260/446 in 201B7, Figure 2G; 54.8% or 292/533 in D1-1; and 52.6% or 214/407 in D1-6, Additional file 4B). We next examined Na_v_1.1 expression differences among the subtypes of GABAergic neurons based on co-expression of PV, calretinin, or somatostatin. In mouse brain, strong Na_v_1.1 expression has been shown in PV-positive interneurons, whereas somatostatin- and calretinin-positive neurons show none [29]. This study produced several calretinin-positive 201B7 control neurons that also stained for Na_v_1.1 after 33 days of differentiation (44.4% or 8/18, Additional file 5A). Somatostatin-positive neurons, on the other hand, presented with either faint or negligible Na_v_1.1-staining in all cases (*N* = 10 and 7 in 201B7 and D1-6 neurons, respectively – Additional file 5B). PV expression was not detectable, even after treatment with sonic-hedgehog (shh) [30] or purmorphamine (a shh-signaling agonist) [31] for ventralization, and/or BMP4 [32] However, we did detect PV mRNA (Additional file 6) as well as mRNA for Nkx2.1, a medial ganglionic eminence neuron marker [33,34] that is elevated by the ventralizing treatments (Additional file 7) [35,36]. Thus, while PV-neuron precursors were likely present, our culture conditions may have interfered with further maturation.

Although it was technically difficult to distinguish subtypes other than GABAergic amongst the Na_v_1.1-positive neurons, some were positive for VGlut1, a marker of glutamatergic neurons, as a minor population (<1%), and on occasion, these neurons co-localized with *SCN1A*-Venus fluorescence (i.e. *SCN1A* expression, Figure 2H and Additional file 4C).

### *SCN1A*-reporter for electrophysiology

**Figure 3:**
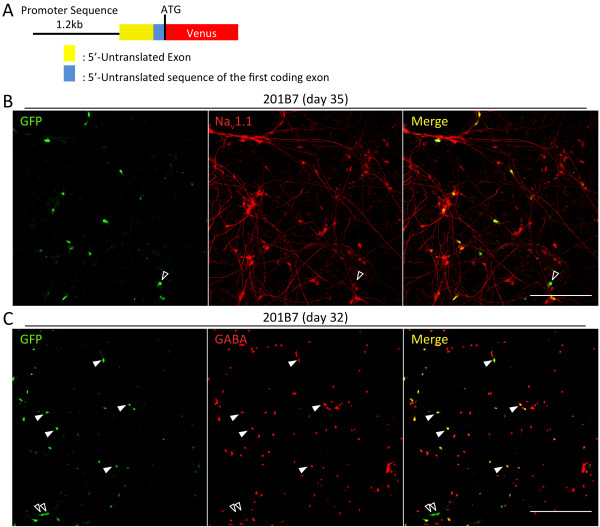
**Structure and characterization of the lentiviral *SCN1A*-reporter used in the electrophysiological analyses.** (**A**) The reporter comprised (5^′^ to 3^′^) a 1.2-kb upstream sequence, a 5^′^-untranslated exon, the 5^′^-end of the first coding exon, and, following the ATG start codon, Venus cDNA. (**B**) & (**C**) 201B7 neurons labeled for Venus (using a GFP antibody) and Na_v_1.1 (**B**) or GABA (**C**). (**B**) Open arrowheads indicate GFP-pseudopositive neurons lacking Na_v_1.1 staining. (**C**) GFP-positive neurons with (arrowhead) and without GABA staining (open arrowhead). Scale bars: 200 μm.

To reliably identify *SCN1A*-expressing neurons for electrophysiological analyses, we generated a lentiviral reporter for *SCN1A*. The reporter contained *SCN1A* promoter sequence [37], some *SCN1A* 5^′^-untranslated region, as well as Venus cDNA following the ATG start codon (Figure 3A). This *SCN1A*-Venus construct was used to infect freshly plated cells from dissociated neurospheres. After several days of differentiation, *SCN1A*-Venus fluorescence developed in a few neurons, which further increased both, in the number of Venus-positive neurons and fluorescence intensity, as neuronal differentiation proceeded.

To confirm co-existence of Na_v_1.1 and Venus protein in the same cells, we employed immunostaining and found that most of the Venus-positive neurons also expressed Na_v_1.1 protein (81% or 81/98 in 201B7 neurons, Figure 3B; 90.1% or 100/111 in D1-1; and 78.8% or 93/118 in D1-6, Additional file 8A). Furthermore, many *SCN1A*-Venus-positive neurons were also positive for GABA (79.3% or 69/87 in 201B7 neurons, Figure 3C; 83.0% or 186/224 in D1-1 neurons; and 70.3% or 152/216 in D1-6, Additional file 8B), indicating GABAergic neurons.

### Neuron selection for electrophysiology

To examine the electrophysiological behavior of control and patient-derived neurons, we conducted current-clamp experiments on cells 22–50 days into neuronal differentiation; shorter differentiation times produced unreliable responses suggesting that the neurons had not fully matured. Hence, neuron selection for electrophysiological analysis was based on the following conditions: (1) clear *SCN1A*-Venus fluorescence; (2) mature neuronal morphology with a large and complex cell body and growth of ≥4 neurites; (3) ≥30 pF membrane capacitance; and (4) resting membrane potential at or more negative than -30 mV.

Based on these criteria, a total of 48 and 27 neurons were recruited for patient-derived cell lines D1-1 and D1-6, respectively; 33 neurons were examined for the 201B7 control cell line. We first established cell capacitance and the resting membrane potential for all cells as indicators for neuron maturity in an effort to minimize inclusion of potentially inappropriate cell responses (Additional file 9A). We found that the resting membrane potential averagely fell between -40 and -45 mV without any statistically discernible difference between the cell lines. The neurons had membrane capacitance mostly up to 70 pF. Some outliers of 100+ pF were also present, but they required excessive current injection to generate action potentials (Additional file 10), which prompted us to remove them from our analyses.

We next examined action potential generation in the current clamp configuration, using 10-ms depolarizing current injections from a holding potential of -70-mV, and we found no statistical difference between patient-derived and control neurons in terms of firing threshold and peak voltage (Additional file 9B).

We then determined the input–output relationship using sustained 500-ms injections of depolarizing current to trigger action potentials. In all cases, the number of action potentials per 500-ms stimulation period increased with the intensity of the injected current. However, as current injection intensified, *amplitude attenuation* became apparent (Additional file 11A). This intensified up to a certain current injection level, where action potentials not only obviously declined in amplitude but also in number, to ultimately stop completely (*depolarization block*, Additional file 11B). Because depolarization block was common (Additional file 12), we suspected that electrically immature neurons were abundant among the cells we had selected for analysis. For further electrophysiological characterization, we therefore admitted neurons only, if they produced 10 or more action potentials.

### Action potential analysis

**Figure 4:**
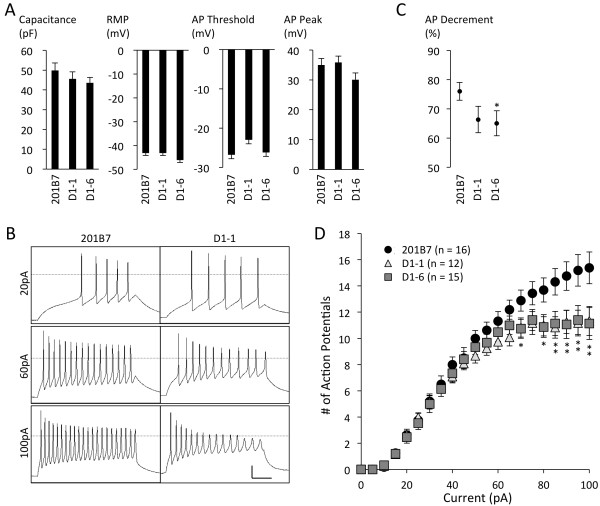
**Electrophysiological characteristics of mature iPSC-derived neurons.** (**A**) Capacitance, resting membrane potential (RMP), action potential (AP) firing threshold, and voltage peak were identical across all neurons analyzed (*P* >0.05, Kruskal-Wallis test); error bars indicate S.E.M. (**B**) Representative traces of AP trains triggered by a 500-ms depolarizing current at the indicated intensities. Transverse dotted lines demark 0 mV membrane potential. Scale bars: 20 mV *vs.* 100 ms. (**C**) Action potential (AP) decrement at current intensities triggering >10 APs calculated as a percentage: 10^th^/1^st^ AP amplitude. Control *vs*. D1-1 (*P* = 0.078) and D1-6 (^*^P = 0.045, ANOVA); D1-1 *vs.* D1-6 (*P* = 0.839) (**D**) Total number of APs during the 500-ms stimulation period *vs.* current injection intensity. When exposed to strong current injections, both patient-derived cell lines produced significantly fewer APs compared to the control (the slope of AP numbers at ≥50 pA, *P* = 0.0102 and 0.0011 for D1-1 and D1-6, respectively, ANCOVA, ^*^P <0.05 for D1-6 only, ^**^P <0.05 for both D1-1 and D1-6, Wilcoxon rank-sum test).

The numbers of neurons for electrophysiological comparisons between cell lines were 12 in D1-1, 15 in D1-6, and 16 in 201B7. Capacitance, resting membrane potential, action potential threshold, and action potential peak voltage in control and patient-derived neurons were statistically indistinguishable (Figure 4A). However, most notably in the input–output relationship, both patient-derived neuron cell lines frequently produced marked amplitude attenuation, which was not seen in control neurons (Figure 4B–C). Furthermore, both Dravet neuron cell lines showed a similar reduction in action potential firing at >50 pA, which, too, was never observed in control cells (Figure 4D). Data of individual neurons (Additional file 13) show that a higher number of D1-1 (33.3% or 4/12) and D1-6 (46.7% or 7/15) neurons reached their peak output prior to reaching a current of 100 pA compared to 201B7 (12.5% or 2/16) neurons. These differences support functional impairment in the patient-derived neurons, especially in GABAergic neurons. The essence is a reduced output capacity during intense stimulation.

## Discussion

In this study, we report on the generation of neurons from DS patient iPSCs. Gene expression and immunocytochemical analyses demonstrated that the control and the two patient cell lines contained neurons of identical character. Electrophysiological analysis of the patient-derived cells revealed impairments in action potential generation in response to sustained current injection, especially with higher current intensities. Specifically, patient-derived iPSC neurons produced fewer action potentials with attenuated amplitudes and earlier depolarization block compared to control neurons. These results are reminiscent of the voltage responses seen in neurons isolated from rodent epilepsy models with *SCN1A* defects [8,9,17,38] and they are consistent with DS pathophysiology that includes an inability of neurons to adequately respond to high-intensity stimulation. Although it was technically difficult to conclusively determine whether the Na_v_1.1-positive neurons were GABAergic or glutamatergic (likely due to cell population heterogeneity and low marker protein expression), data from immunocytochemical analyses suggest that the Na_v_1.1-positive neurons were mostly GABAergic. Furthermore, the majority of *SCN1A* Venus-positive neurons showed GABA immunostaining, which supports that the neurons undergoing electrophysiological analysis in this study were phenotypically homogeneous. We therefore interpreted our findings in the context of a functional decline in GABAergic neuron activity – defective inhibition. Of course, we cannot exclude involvement of other neuron types. In the context of the data presented here, however, it is possible that the pathophysiology of human and mouse Dravet syndrome employs similar mechanisms.

Several differences may exist between human and rodent brains with respect to Na_v_1.1 expression. In rodent cerebral cortex, Na_v_1.1 is predominantly expressed in the axon initial segment of GABAergic interneurons. Pyramidal neurons also express Na_v_1.1 [8,39,40], albeit at a minor level [9,15]. Furthermore, epilepsy models with *SCN1A* defects have identified functional deficits in GABAergic interneurons, but not in pyramidal neurons [8,9,41]. In human brain, Na_v_1.1 expression differs from what is seen in rodents: Na_v_1.1 shows somatodendritic localization and expression in pyramidal neurons, specifically in cortical layer V and in the hippocampus [42-44]. This may be attributed to different experimental conditions and antibodies used, but, if rodent and human expression patterns indeed diverge, then it is conceivable that the associated pathophysiology differs as well. Pyramidal neurons use glutamate as their neurotransmitter, and our analyses showed that iPSCs-derived neurons expressing *SCN1A*-Venus were also positive for VGlut1. Unfortunately, the culture conditions in our study did not permit ready differentiation into glutamatergic neurons, which kept their number below what is usable for functional analyses. Functional characterization of non-GABAergic neurons must be addressed in the future to enhance our understanding of this DS model, and possibly unveil further pathogenic mechanisms.

With current methodology, establishing iPSC lines is labor- and time- consuming. Future research into human *in vitro* disease models may soon overcome these obstacles as other sources of pluripotent stem cells are considered, such as Nestin-expressing hair follicle stem cells [45,46]. They are easily accessible, they can be utilized without any genetic manipulation, and they have the potential to differentiate into neurons. If stable and efficient neural induction and maturation methods are established, for *in vivo* neuronal disease modeling will be possible.

## Conclusions

With this study, we report the first successful generation of a human-based *in vitro* DS model. Our data are consistent with a functional decline in GABAergic neurons, which may contribute to DS epileptogenesis. The results are encouraging that patient-derived iPSC models can be utilized in human epilepsy research. They may, in fact, provide unparalleled insight into pathogenic mechanisms, and a uniquely suited research platform for drug development.

## Materials and methods

### Isolation of human skin fibroblasts and generation of iPSCs

Skin fibroblasts were isolated from a skin punch biopsy of the patient’s upper arm with the approval by the Human Ethics Committee of Fukuoka University (Approval No. 361). The parents of the patient provided signed informed consent before the study. Fibroblasts were cultured in DMEM containing 10% fetal bovine serum, 50 IU/mL penicillin, and 50 mg/mL streptomycin. The generation, maintenance, and characterization of iPSCs were performed as previously described [26,47]. Briefly, fibroblasts were lentivirus-trunsduced with Slc7a1 and plated at a density of 3.5 × 10^5^ cells/60-mm dish. The next day, 4 reprogramming factors (Sox2, Klf4, Oct3/4, and c-Myc) were transduced using retroviruses. Seven days thereafter, the fibroblasts were re-plated at a density of 5 × 10^3^–5 × 10^5^ cells/100-mm dish with a mitomycin C-treated SNL feeder layer. The SNL feeder cells were obtained from the Wellcome Trust Sanger Institute (Hinxton, England). The next day, the medium was replaced with human iPS medium, which was DMEM/F12 containing 20% knockout serum replacement, 2 mM L-glutamine, 0.1 mM nonessential amino acids, 0.1 mM 2-mercaptoethanol, 4 μg/mL basic FGF (R&D Systems, Minneapolis, MN), 50 IU/mL penicillin, and 50 mg/mL streptomycin. The medium was changed either daily or every other day until iPSC colonies were isolated, 24–28 days from the transduction of the reprogramming factors. iPSC clones were selected based on reprogramming transgene silencing (real-time PCR), colony morphology, expression of markers for undifferentiated cells (immunocytochemistry), pluripotency (teratoma formation), and neural induction efficiency. For iPSC maintenance, the medium was changed daily, and the cells passaged every 4–7 days depending on colony size. iPSCs passaged < 32 times were used for neural induction. Details on the PCR conditions for *SCN1A*, *SCN2A*, *SCN1B*, and *SCN2B* sequencing are available on request. The control iPSCs, 201B7, were provided by the RIKEN BioResource Center through the Project for Realization of Regenerative Medicine and the National Bio-Resource Project of the Ministry of Education, Culture, Sports, Science & Technology (MEXT) in Japan. iPSC production was approved by the Keio University School of Medicine Ethics Committee (Approval No. 20-16-18) and the Human Ethics Committee of Fukuoka University (Approval No. 361).

### Neuronal differentiation of human iPSCs

Neuronal differentiation of human iPSCs was performed as previously described [48] with minor modifications [19,22,49]. Briefly, iPSC colonies were detached from feeder layers, and were cultured in suspension as embryoid bodies (EBs) for about 30 days in bacteriologic dishes (Kord-Valmark/Myers, Akron, OH). For neurosphere formation, EBs were enzymatically dissociated and the single cells were cultured in suspension in the serum-free neurosphere medium (media-hormone-mix) for 10 to 14 days. The resulting neurospheres could be repeatedly passaged using the dissociation procedures described above. Neurospheres passaged 1–3 times were used for analysis. For ventralization of neuronal properties in some preliminary assays, 5 or 30 nM of sonic hedgehog (R&D Systems, Minneapolis, MN) or 1 μM of purmorphamine (EMD Millipore, Billerica, MA) were added to the medium during the later phase of embryoid body and/or neurosphere formation. For terminal differentiation, neurospheres were plated onto poly-l-ornithine/fibronectin-coated coverslips in a media-hormone-mix supplemented with B27 supplement. Dissociated cells were plated at an approximate density of 1 × 10^5^ cells/cm^2^. Both dissociated and undissociated cells were cultured for varying periods depending on the assay. To enhance cell viability and to promote neuronal maturation, the differentiation medium was supplemented as follows: 10 ng/mL rhBDNF and rhGDNF (R&D Systems), and 200 μg/mL L-ascorbic acid (Sigma-Aldrich, St. Louis, MO). Attempts to induce parvalbumin-positive neurons were made with 100 ng/mL of BMP4 (R&D Systems) on day 10 of neuronal differentiation and continued until the assay was conducted.

### RNA isolation, reverse transcription, and real-time PCR analysis

Total cellular RNA was extracted using TRIZOL Reagent (Life Technologies, Carlsbad, CA), RNase-Free DNase Set (Qiagen, Venlo, Netherlands), and RNeasy Mini or Micro Kits (Qiagen). Complimentary DNA synthesis was performed using the SuperScript III First-Strand Synthesis System for RT-PCR (Life Technologies) with oligo-dT primers from 0.2–1.0 μg of total RNA, according to the manufacturer’s guidelines. To analyze the relative expression of different mRNAs, the amount of cDNA was normalized to β-actin mRNA expression. The mRNA expression levels in iPSC-derived neurons were determined from at least three separately cultivated samples. Real-time PCR was performed using the LightCycler 480 System II (Roche Diagnostics, Basel, Switzerland) with the SYBR Premix Ex Taq (Takara Bio, Shiga, Japan). Primer sequences for real-time PCR are listed in Table 1.

**Table 1:** Primer details for real-time PCR

**Target**	**Forward**	**Reverse**
*β-actin*	GATCAAGATCATTGCTCCTCCT	GGGTGTAACGCAACTAAGTCA
Sox2 (tg^*^)	ACGGCCATTAACGGCACACTG	CCCTTTTTCTGGAGACTAAATAAA
*Klf4 (tg)*	CACCTCGCCTTACACATGAAGAG	
*Oct3/4 (tg)*	TCTGGGCTCTCCCATGCATTCAAAC	
*c-Myc (tg)*	CTTGAACAGCTACGGAACTCTTG	
*SCN1A*	AACAGAATCAGGCCACCTTG	CACTGGGCTCTCTGGAATG
*SCN2A*	GCTACACGAGCTTTGACACC	CCCAAGAAAATGACCAGCAC
*SCN3A*	ATGGTGTGGTTTCCTTGGTG	TGACTTCCGTTTCTGTGGTG
*SCN8A*	GGACCCATGGAACTGGTTAG	ACCCTGAAAGTGCGTAGAGC
*Nkx2.1*	AGCACACGACTCCGTTCTCA	CCCTCCATGCCCACTTTCTT
*Parvalbumin*	CTGGACAAGGACAAAAGTGG	ACAGGTCTCTGGCATCTGG

All sequences are displayed in 5′-to-3′ direction. ^*^tg, transgene.

### Immunocytochemistry

Cells on coverslips were fixed with 4% paraformaldehyde for 10–30 min at room temperature, followed by washing 3 times with PBS. After incubating with blocking buffer (PBS containing 5% normal goat or fetal calf serum and 0.1–0.3% triton X-100) for 1 h at room temperature, the cells were incubated overnight at 4°C with primary antibodies diluted with the blocking buffer. Details of primary antibodies and the dilution conditions are presented below. The cells were then washed 3 times with PBS and incubated with secondary antibodies conjugated with Alexa Fluor 488 or Alexa Fluor 555 (1:500, Life Technologies) and Hoechst33342 (2 μg/mL, Dojindo Laboratories, Kumamoto, Japan) for 1 h at room temperature. After washing 3 times with PBS and a single wash with distilled water, the coverslips were mounted on slides with FluorSave Reagent (EMD Millipore/Merck Group). Images were acquired using a confocal laser-scanning microscope, FV1000-D (Olympus, Tokyo, Japan). Observation through 20× objective was used to determine whether Na_v_1.1-positive neurons were also positive for GAD67, calretinin, or GFP (for detection of Venus).

### Primary antibodies used in immunocytochemistry

Anti-Na_v_1.1 (rabbit IgG, 1:500, Cat No. ASC-001 – Alomone Labs, Israel). This antibody targets the peptide TASEHSREPSAAGRLSD, which corresponds to amino acids 465–481 in the internal D1–D2 linker human full-length Na_v_1.1 (Reference sequence: NP_001159435.1). Anti-Sodium Channel ‘PAN’ (mouse IgG_1_, 1:100, Cat No. S8809 – Sigma-Aldrich, St. Louis, MO) targets the peptide TEEQKKYYNAMKKLGSKK in the intracellular D3–D4 linker of Na_v_ channels which is identical in all known vertebrate Na_v_ channel isoforms. Anti-SSEA1 (mouse IgM, 1:500, Cat No. ab16285 – Abcam, Cambridge, MA), anti-SSEA4 (mouse IgG3, 1:500, ab16287 – Abcam), anti-TRA-1-60 (mouse IgM, 1:1000, Cat No. MAB4369 – EMD Millipore, Billerica, MA USA), anti-TRA-1-81 (mouse IgM, 1:1000, MAB4381 – EMD Millipore), anti-Oct3/4 (Rb IgG, 1:500, Cat No. sc-9081 – Santa Cruz Biotechnology, Santa Cruz, CA), anti-Nanog (rabbit IgG, 1:100, Cat No.RCAB0001P – Cosmo Bio, Carlsbad, CA), anti-βIII-tubulin (mouse IgG_2b_, 1:1000, Cat No. T8660 – Sigma-Aldrich, St. Louis, MO), anti-GFAP (rabbit IgG, 1:4000, Cat No. Z0334 – Dako, Denmark), anti-GFP (mouse IgG_2a_, 1:100, A11120 – Life Technologies, Carlsbad, CA, or rabbit IgG, 1:500, gift from Dr. Y. Misumi, Fukuoka University), anti-Ankyrin G (mouse IgG_1_, 1:100, 33–8800 – Life Technologies), anti-GAD67 (mouse IgG_2a_, 1:2000, MAB5406 – EMD Millipore), anti-Parvalbumin (mouse IgG_1_, 1:1000, MAB1572 – EMD Millipore), anti-Somatostatin (Rat IgG_2b_, 1:100, ab30788 – Abcam), anti-Calretinin (mouse, 1:1000, Cat No. 6B3 – Swant, Switzerland), anti-VGlut1 (rabbit IgG, 1:1000, Cat No. 135303 – Synaptic Systems, Germany), and anti-GABA (rabbit IgG, 1:2000, A2052– Sigma-Aldrich).

### Generation of *SCN1A* reporter lentivirus

The upstream genomic sequence of an *SCN1A* 5^′^-untranslated exon (previously referred to as “h1b” by Martin, et al.[50], or “hB” by Nakayama, et al.[37]) was used as *SCN1A* promoter sequence. The 1,200-bp sequence stretch showed strong promoter activity and was obtained from the patient’s genomic DNA. Aforementioned untranslated exon connected with the 5^′^-end of the first coding exon, which was obtained from D1-1 iPSC-derived neuronal cDNA. These fragments were connected via PCR and transferred into the pSIN-Venus vector, which has a cloning site connected to Venus cDNA (constructed by Y. Okada, Keio University). For lentivirus production, the pSIN construct, pLP1, pLP2, and pLP/VSVG plasmids (Life Technologies) were mixed and transfected into 293FT cells using CalPhos Mammalian Transfection Kit (Clontech/Takara Bio) or Lipofectamine 2000 Reagent (Life Technologies). The medium was changed the following day. Two days thereafter, the virus-containing medium was collected, filtered, and ultracentrifuged at 25,000 rpm with an SW 28 Rotor (Beckman Coulter, Brea, CA USA), at 4°C for 90 min. The viral pellet was resuspended in 1/200 of the original medium volume with media-hormone-mix, aliquoted, and stored at -80°C until use.

### Electrophysiological analysis

Electrophysiological analysis employed room temperature current-clamping of iPSC-derived neurons in the whole-cell configuration. Cell micrographs were produced with an upright microscope (BX51WI – Olympus, Melville, NY) equipped with a CMOS image sensor camera, ORCA-Flash2.8 (Hamamatsu Photonics, Japan). Reporter fluorescence Venus was detected through a 40x water-immersion objective (LUMPlanFI/IR2 – Olympus) with a U-MGFPHQ cube (excitation: 460–480 nm, dichroic mirror: 485 nm, emission: 495–540 nm – Olympus) and processed with Aquacosmos software (Hamamatsu, Japan). The extracellular solution contained 150 mM NaCl, 4 mM KCl, 2 mM CaCl_2_, 2 mM MgCl_2_·6 H_2_O, 10 mM HEPES, and 10 mM glucose adjusted to pH 7.4 with NaOH. Patch pipettes were made from borosilicate glass with filament (Cat No. FB150-86-0 – Sutter Instruments, Novato, CA) and pulled to resistances of 2–4 MΩ (P-97, Sutter Instruments) when filled with 0.22-μm filtered intracellular solution of the following composition: 117 mM K-methanesulfonate, 9 mM EGTA, 9 mM HEPES, 1.8 mM MgCl_2_·6 H_2_O, 29 mM sucrose, 4 mM Mg-ATP, 0.3 mM Tris-GTP, and 5 mM KCl adjusted to pH 7.3 with KOH. Whole-cell patch-clamp recordings were carried out using an Axopatch 700B amplifier (Axon Instruments, Sunnyville, CA) and pCLAMP 10 software (Axon Instruments). Signals were low-pass Bessel filtered at 10 kHz and sampled at a 50 kHz with an Axon Digidata 1440A digitizer (Axon Instruments). Cell capacitance was calculated by integrating the capacitive current evoked by a 10-mV depolarizing pulse from a holding potential of -65 mV. The resting membrane potential was determined from the mean potential during a 10-s continuous recording in zero-current clamp mode. During current-clamp experiments, cells were held at -70 mV by constant current injection, as needed. Single action potentials, operationally defined to minimally reach 0 mV, were evoked by current injection (10 ms) to determine their firing thresholds and peak voltages. The injection current amplitude was increased in 10-pA increments from sub- to supra-threshold. To investigate the input–output relationship, sustained depolarizing currents (500 ms) were injected and the current amplitude was increased from 5 to 100 pA in 5-pA increments. Final data was taken from neurons on at least 8 coverslips of at least 4 separately cultivated samples in each clone. Electrophysiological data were analyzed using pCLAMP 10 software (Axon Instruments, Sunnyvale, CA).

### Statistical analysis

All of the data analyses were performed using SAS (Statistical Analysis System) Software Package (Ver. 9.2, SAS Institute Inc., Cary, NC) at Fukuoka University (Fukuoka Japan). Na_v_ gene expression was compared with one-way ANOVA (between Na_v_ channel genes) and two-way ANOVA (between iPSC clones). Cell capacitance, resting membrane potential, action potential firing threshold, peak voltage, action potential decrement, and area under the input–output relationship curve were compared among the clones using one-way ANOVA (with Scheffe’s *post hoc* test) and/or the Kruskal-Wallis test. The action potential number for each injection level in the input–output relationship was compared between the clones using the Wilcoxon rank-sum test. The slope of the number of action potentials *vs*. injected current in Figure 4C was compared using ANCOVA. Data are presented as mean ± standard error of the mean (S.E.M.), and P-values <0.05 were deemed significant.

## Competing interests

The authors declare that they have no competing interests.

## Authors’ contributions

NH designed, executed, and directed the study, and wrote the paper. TU performed all electrophysiological assays. CL designed the electrophysiological analyses and contributed to the writing of the manuscript. MM and SK assisted with the electrophysiological assay methods and helped with data interpretation. YM contributed to the immunocytochemical analysis. YO, YI and WA contributed to all cell culture and biologic assay procedures. KN performed the pathologic analysis of the iPSC-derived teratomas. BZ performed statistical analysis. YS contributed to clinical and genetic analysis of the patient. HO and SH coordinated the study. All authors read and approved the final manuscript.

## Supplementary Material

Additional file 1 **Schematic representation of Na**_**v**_**1.1 topology.** The typical Na_v_ channel complex consists of one main, pore-forming α subunit (Na_v_1.1–Na_v_1.9) and one or more auxiliary β subunits. The α subunit is made up of four homologous domains (D1–D4) with six transmembrane regions each (S1–S6). Voltage sensitivity is mediated by positively charged residues in the S4 regions that move in the electrical field upon depolarization to cause a conformational change that favors opening of the channel. The antigenic regions for the Na_v_ antibodies are shown as grey boxes; the site of the truncating mutation in D4/S4 is highlighted in red. Terminated at the R1645 residue, the Na_v_1.1 protein looses the faded protein portion (i.e., part of the voltage sensor, pore-lining residues and the entire C- terminus) and thereby its ability to function. Click here for file

Additional file 2 **Real-time PCR efficiency, Na**_**v **_**genes.** Click here for file

Additional file 3 **Na**_**v**_**1.1 antibody selectivity.** Representative images of Na_v_1.1 immunostaining (red). The image on the right was acquired after the Na_v_1.1 antibody had been pre-treated with epitope peptide. Scale bar, 50 μm. Nuclei are stained blue with Hoechst33342 to facilitate cell identification. Click here for file

Additional file 4 **Characterization of patient-derived neurons.** (A) Intense expressions of PAN-Na_v_ in the axon initial segment (solid arrowheads) of *SCN1A* Venus-positive neurons. (B) Co-localization of Na_v_1.1 and GAD67 staining. (C) VGlut1-positive neuron co-localized with *SCN1A* Venus (solid arrowheads). Scale bars: 30 μm (A), 200 μm (B), and 50 μm (C). Click here for file

Additional file 5 **Characterization of Na**_**v**_**1.1-positive neurons.** (a) Calretinin-positive neurons with (arrowhead) and without Na_v_1.1 staining (open arrowhead). Scale bar, 50 μm. (b) Somatostatin-positive neurons are negative for Na_v_1.1. Scale bar, 100 μm. Click here for file

Additional file 6 **RT-PCR of parvalbumin mRNA from iPSCs-derived neurons.** 180-bp bands are indicated beta-actin mRNA expression. 85-bp bands demark parvalbumin (PV). When total RNA was used as template (RT-), no product was generated. Click here for file

Additional file 7 **Increase in Nkx2.1 mRNA expression following treatment with sonic hedgehog (SHH) or purmorphamine.** (a) During embryoid body formation (approx. 20–30 days) of cell line D1-1, the growth medium was supplemented with SHH to the indicated concentrations. This resulted in a dose-related increase in Nkx2.1 mRNA expression. Data from two different setups were averaged and normalized to the control (0 nM SHH); error bars are S.E.M. (b) Similar setup as in Panel (a), but SHH was added during neurosphere (NS) formation; cell line D1-6. This produced an increase in Nkx2.1 mRNA expression, although apparently not in dose-dependent fashion, which may relate to SHH only maintaining Nkx2.1 expression rather than inducing new ventral neuronal precursors. (c) Setup similar to Panel (a), albeit with purmorphamine treatment. Click here for file

Additional file 8 **Na**_**v**_**1.1 and GABA expression in *SCN1A *Venus-positive patient neurons.** Venus was detected using a GFP antibody. (A) Venus-positive neurons lacking Na_v_1.1 staining, open arrowheads. (B) Venus-positive neurons with (solid arrowheads) and without GABA staining (open arrowhead). Scale bars: 200 μm. Click here for file

Additional file 9 **Electrophysiological characteristics of all recruited iPSC-derived neurons.** (A) Capacitance & resting membrane potential (RMP) and (B) action potential (AP) firing threshold & voltage peak. No statistical differences were found in all items (*P* >0.05, Kruskal-Wallis test). Error bars indicate S.E.M. Click here for file

Additional file 10 **Input–output relationship of large (≥100 pF) control neurons.** Current clamping as in Figure 4. This produced a set number of action potentials per 500-ms stimulation period, which was plotted against the injected current amplitude. Note the size-dependent increase in the current required to trigger the same number of action potentials compared to smaller neurons (average for Figure 4C depicted in bold). Click here for file

Additional file 11 **Illustration of “*action potential attenuation*” and “*depolarization block*” in current-clamped neurons.** (A) The number of action potentials increased with stronger current injections but a simultaneous tapering of action potential amplitude was apparent. (B) Action potential tapering reached a state where further firing was prevented despite continued stimulation. Rectangular pulses represent current injection periods (500 ms) at the indicated intensities. Click here for file

Additional file 12 **Example current-clamp traces of 201B7 control neurons with immature voltage responses.** 500-ms depolarizing currents were injected at the indicated intensities. Transverse dotted lines demark 0 mV membrane potential. Scale bars, 20 mV *vs*. 100 ms. Click here for file

Additional file 13 **Individual input–output relationship plots for control and Dravet-derived neurons.** Experimental setup and plotting as in Additional file 6. Each line plot represents one cell. Current injections of <100 pA frequently maxed out the number of action potentials triggered in patient neurons (D1-1 and D1-6), but only rarely in the control neurons (201B7). Click here for file
