# Supplementary figures and images for: A human Dravet syndrome model from patient induced pluripotent stem cells
Source: Mol Brain. 2013 May 2;6:19. doi: 10.1186/1756-6606-6-19 (PMC3655893; doi:10.1186/1756-6606-6-19)

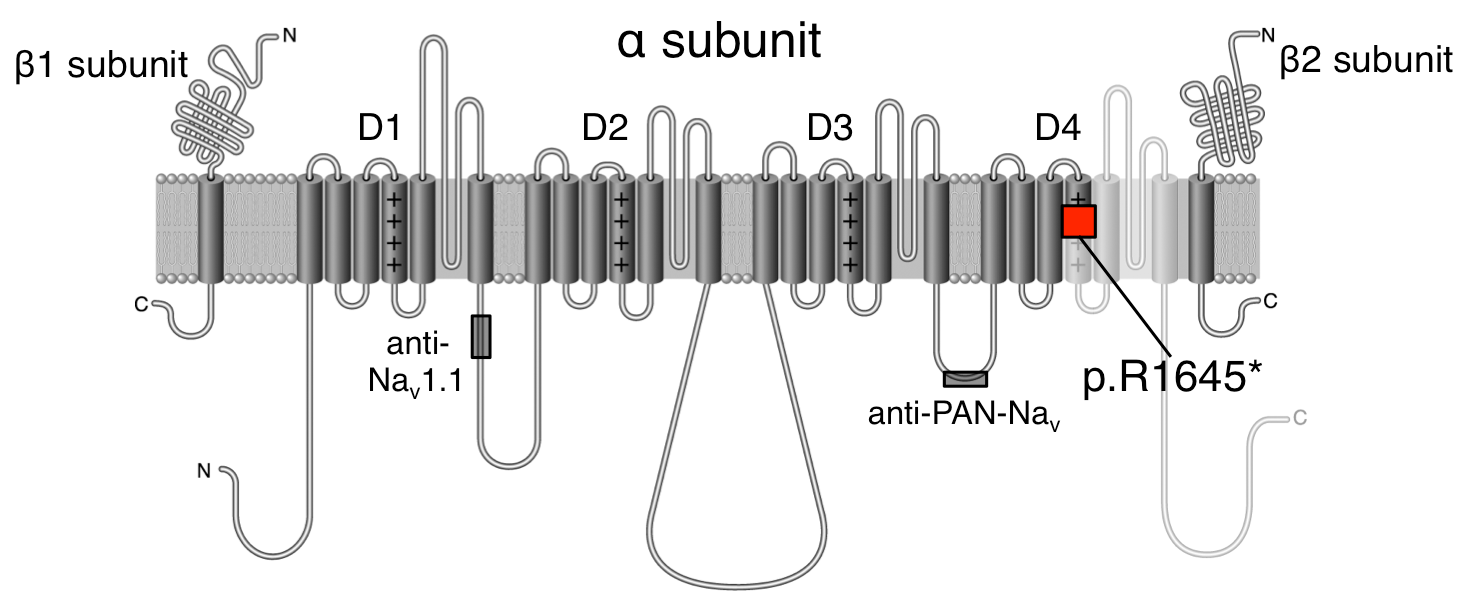

Supplement: Additional file 1 — Schematic representation of Nav1.1 topology. The typical Nav channel complex consists of one main, pore-forming α subunit (Nav1.1–Nav1.9) and one or more auxiliary β subunits. The α subunit is made up of four homologous domains (D1–D4) with six transmembrane regions each (S1–S6). Voltage sensitivity is mediated by positively charged residues in the S4 regions that move in the electrical field upon depolarization to cause a conformational change that favors opening of the channel. The antigenic regions for the Nav antibodies are shown as grey boxes; the site of the truncating mutation in D4/S4 is highlighted in red. Terminated at the R1645 residue, the Nav1.1 protein looses the faded protein portion (i.e., part of the voltage sensor, pore-lining residues and the entire C- terminus) and thereby its ability to function. [file 1756-6606-6-19-S1.tiff]

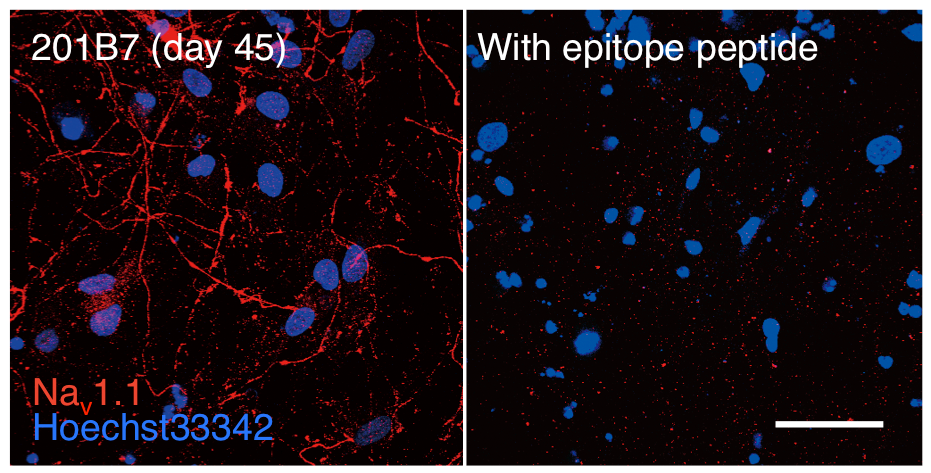

Supplement: Additional file 3 — Nav1.1 antibody selectivity. Representative images of Nav1.1 immunostaining (red). The image on the right was acquired after the Nav1.1 antibody had been pre-treated with epitope peptide. Scale bar, 50 μm. Nuclei are stained blue with Hoechst33342 to facilitate cell identification. [file 1756-6606-6-19-S3.tiff]

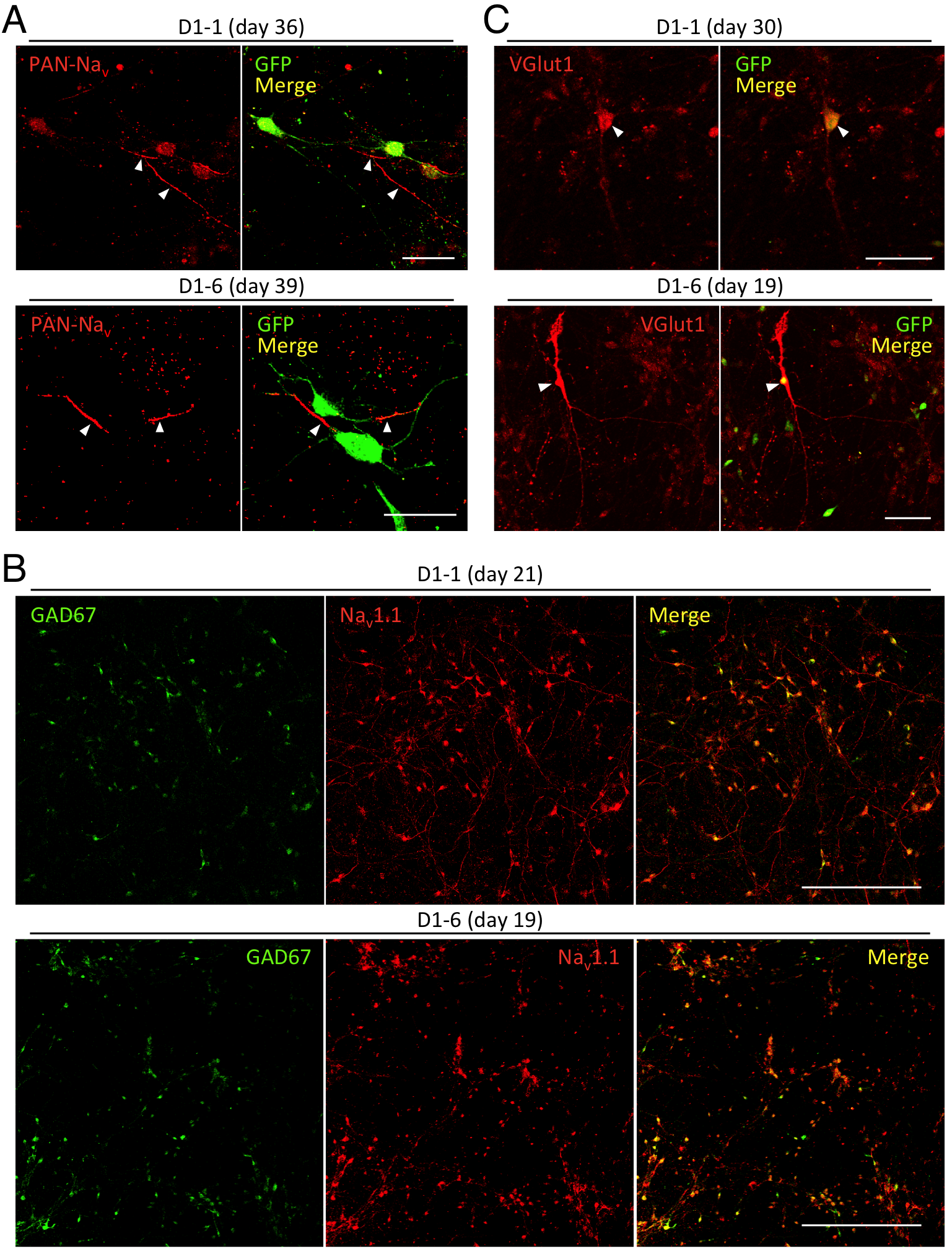

Supplement: Additional file 4 — Characterization of patient-derived neurons. (A) Intense expressions of PAN-Nav in the axon initial segment (solid arrowheads) of SCN1A Venus-positive neurons. (B) Co-localization of Nav1.1 and GAD67 staining. (C) VGlut1-positive neuron co-localized with SCN1A Venus (solid arrowheads). Scale bars: 30 μm (A), 200 μm (B), and 50 μm (C). [file 1756-6606-6-19-S4.tiff]

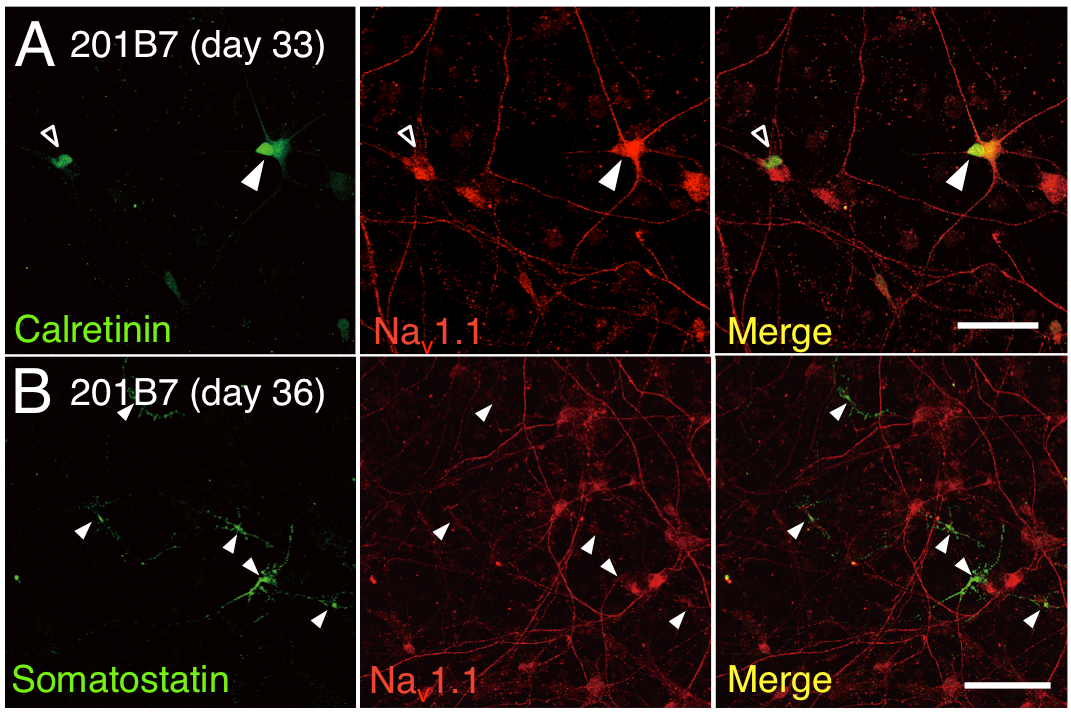

Supplement: Additional file 5 — Characterization of Nav1.1-positive neurons. (a) Calretinin-positive neurons with (arrowhead) and without Nav1.1 staining (open arrowhead). Scale bar, 50 μm. (b) Somatostatin-positive neurons are negative for Nav1.1. Scale bar, 100 μm. [file 1756-6606-6-19-S5.tiff]

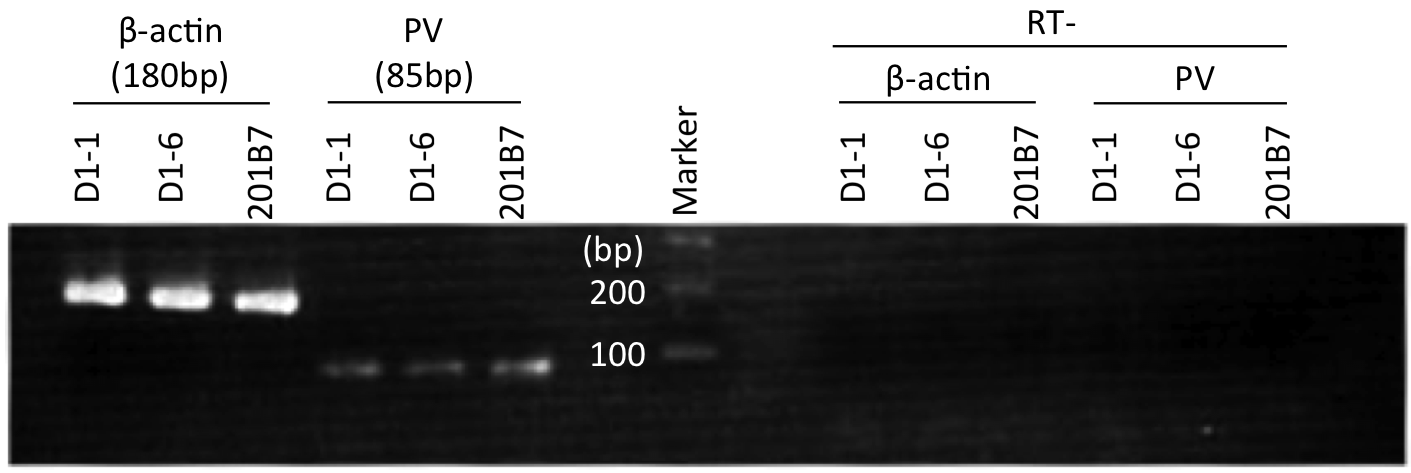

Supplement: Additional file 6 — RT-PCR of parvalbumin mRNA from iPSCs-derived neurons. 180-bp bands are indicated beta-actin mRNA expression. 85-bp bands demark parvalbumin (PV). When total RNA was used as template (RT-), no product was generated. [file 1756-6606-6-19-S6.tiff]

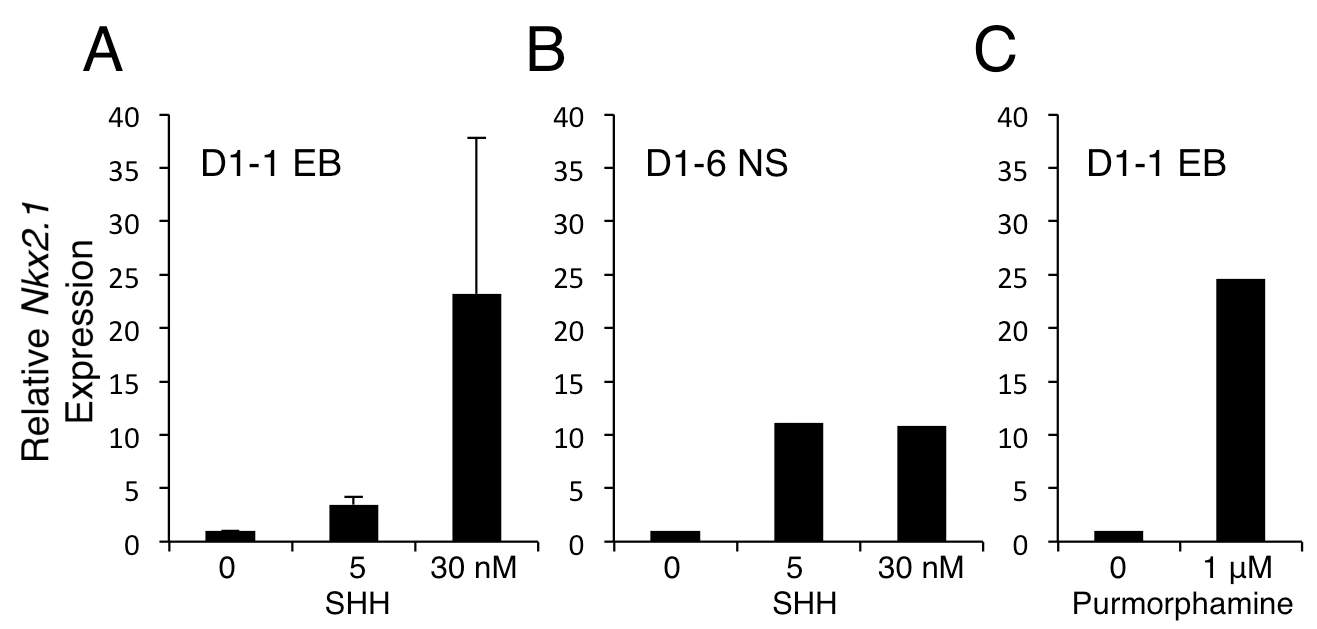

Supplement: Additional file 7 — Increase in Nkx2.1 mRNA expression following treatment with sonic hedgehog (SHH) or purmorphamine. (a) During embryoid body formation (approx. 20–30 days) of cell line D1-1, the growth medium was supplemented with SHH to the indicated concentrations. This resulted in a dose-related increase in Nkx2.1 mRNA expression. Data from two different setups were averaged and normalized to the control (0 nM SHH); error bars are S.E.M. (b) Similar setup as in Panel (a), but SHH was added during neurosphere (NS) formation; cell line D1-6. This produced an increase in Nkx2.1 mRNA expression, although apparently not in dose-dependent fashion, which may relate to SHH only maintaining Nkx2.1 expression rather than inducing new ventral neuronal precursors. (c) Setup similar to Panel (a), albeit with purmorphamine treatment. [file 1756-6606-6-19-S7.tiff]

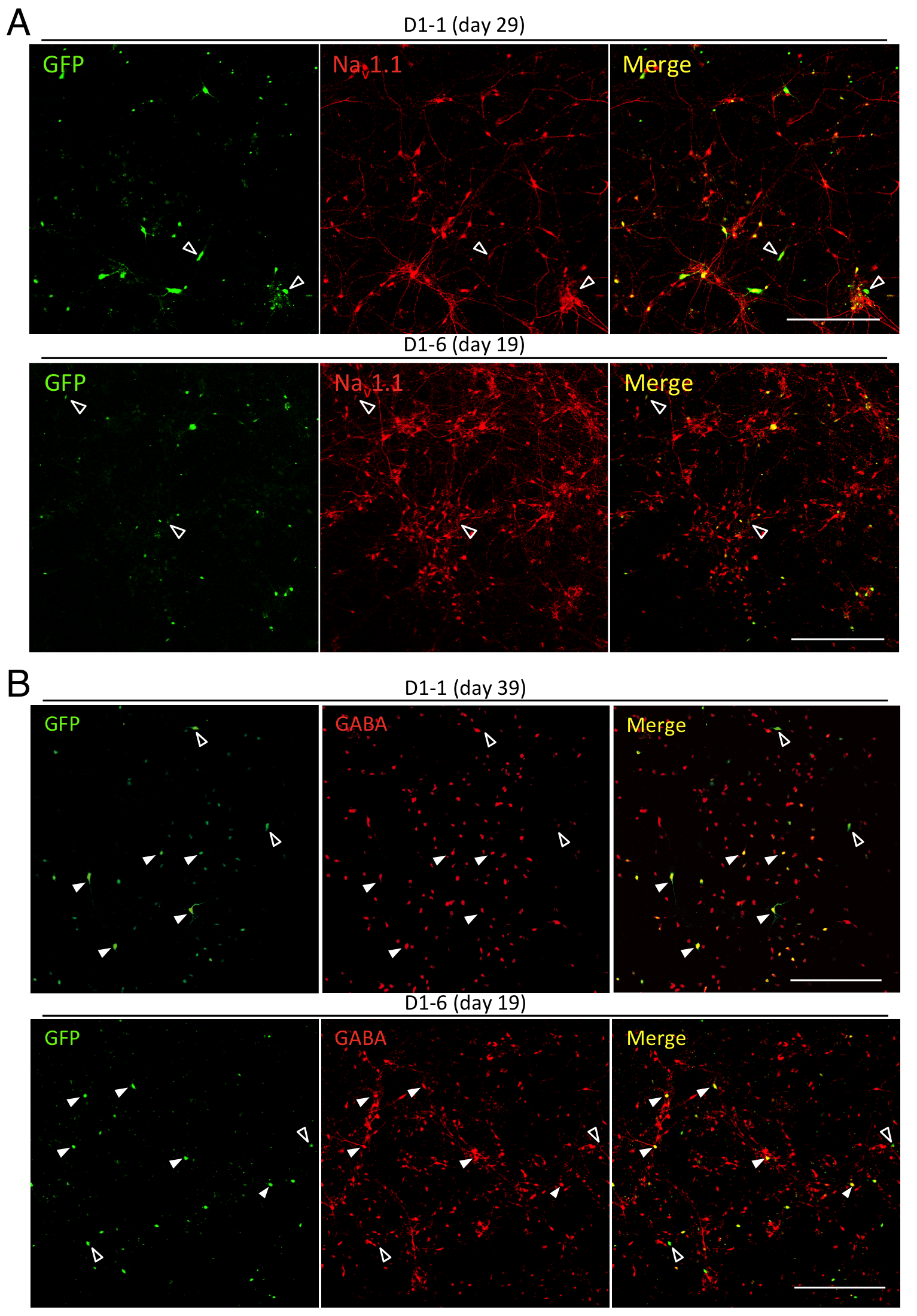

Supplement: Additional file 8 — Nav1.1 and GABA expression in SCN1A Venus-positive patient neurons. Venus was detected using a GFP antibody. (A) Venus-positive neurons lacking Nav1.1 staining, open arrowheads. (B) Venus-positive neurons with (solid arrowheads) and without GABA staining (open arrowhead). Scale bars: 200 μm. [file 1756-6606-6-19-S8.tiff]

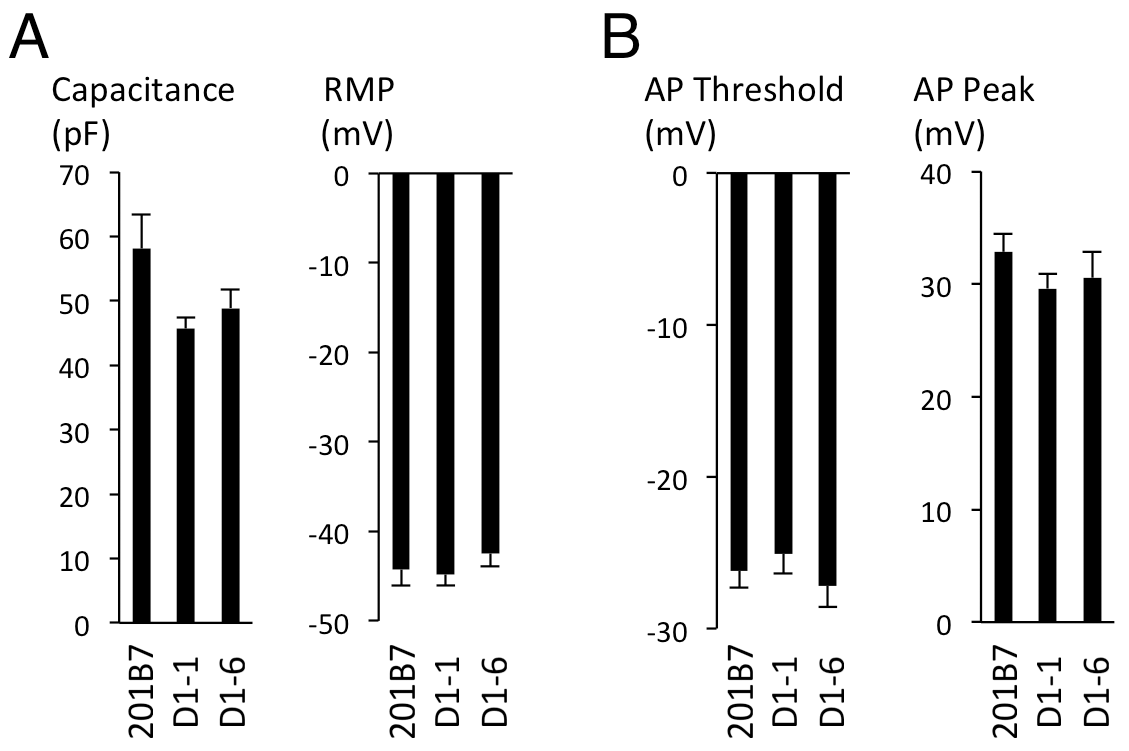

Supplement: Additional file 9 — Electrophysiological characteristics of all recruited iPSC-derived neurons. (A) Capacitance & resting membrane potential (RMP) and (B) action potential (AP) firing threshold & voltage peak. No statistical differences were found in all items (P >0.05, Kruskal-Wallis test). Error bars indicate S.E.M. [file 1756-6606-6-19-S9.tiff]

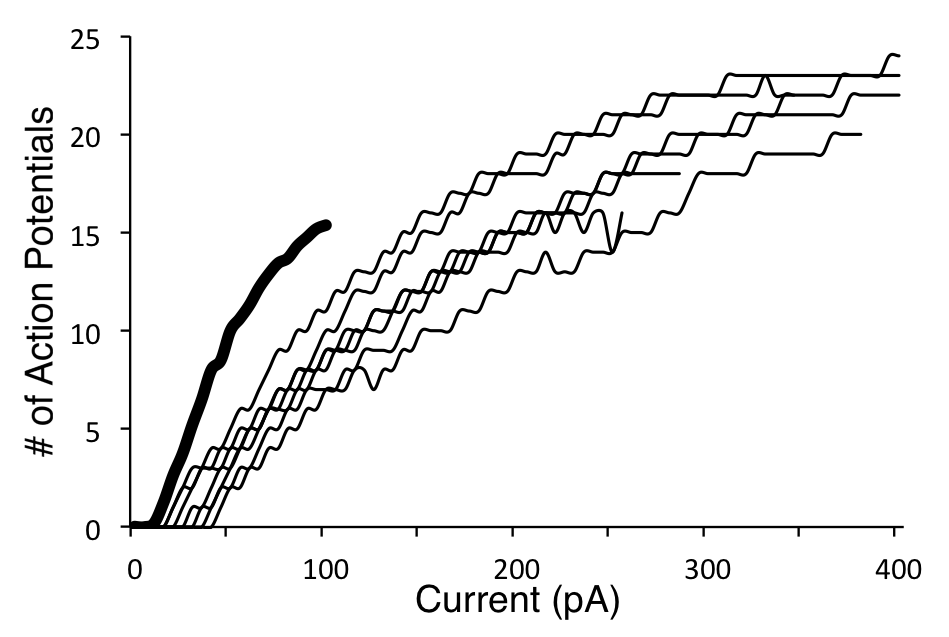

Supplement: Additional file 10 — Input–output relationship of large (≥100 pF) control neurons. Current clamping as in Figure 4. This produced a set number of action potentials per 500-ms stimulation period, which was plotted against the injected current amplitude. Note the size-dependent increase in the current required to trigger the same number of action potentials compared to smaller neurons (average for Figure 4C depicted in bold). [file 1756-6606-6-19-S10.tiff]

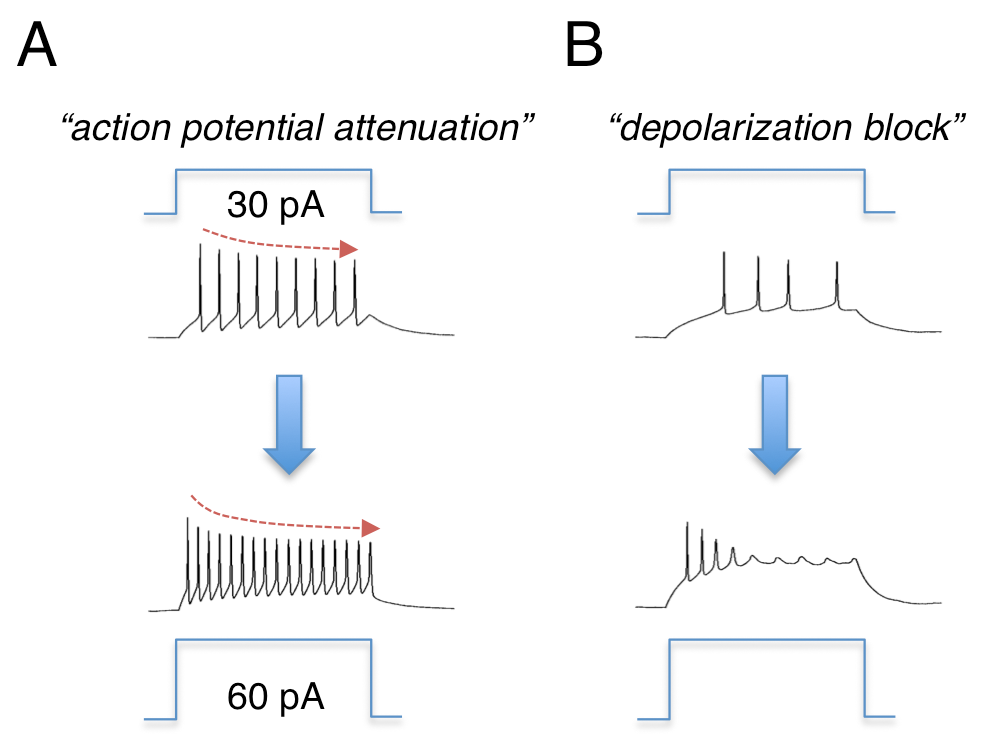

Supplement: Additional file 11 — Illustration of “action potential attenuation” and “depolarization block” in current-clamped neurons. (A) The number of action potentials increased with stronger current injections but a simultaneous tapering of action potential amplitude was apparent. (B) Action potential tapering reached a state where further firing was prevented despite continued stimulation. Rectangular pulses represent current injection periods (500 ms) at the indicated intensities. [file 1756-6606-6-19-S11.tiff]

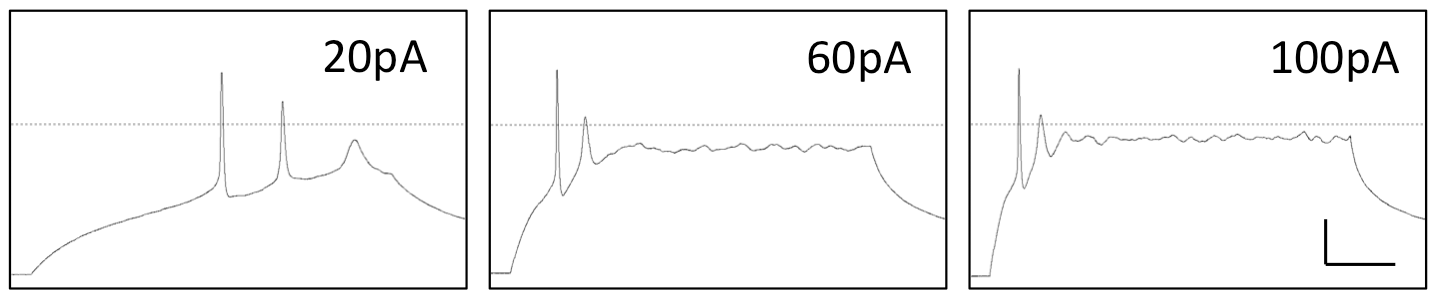

Supplement: Additional file 12 — Example current-clamp traces of 201B7 control neurons with immature voltage responses. 500-ms depolarizing currents were injected at the indicated intensities. Transverse dotted lines demark 0 mV membrane potential. Scale bars, 20 mV vs. 100 ms. [file 1756-6606-6-19-S12.tiff]

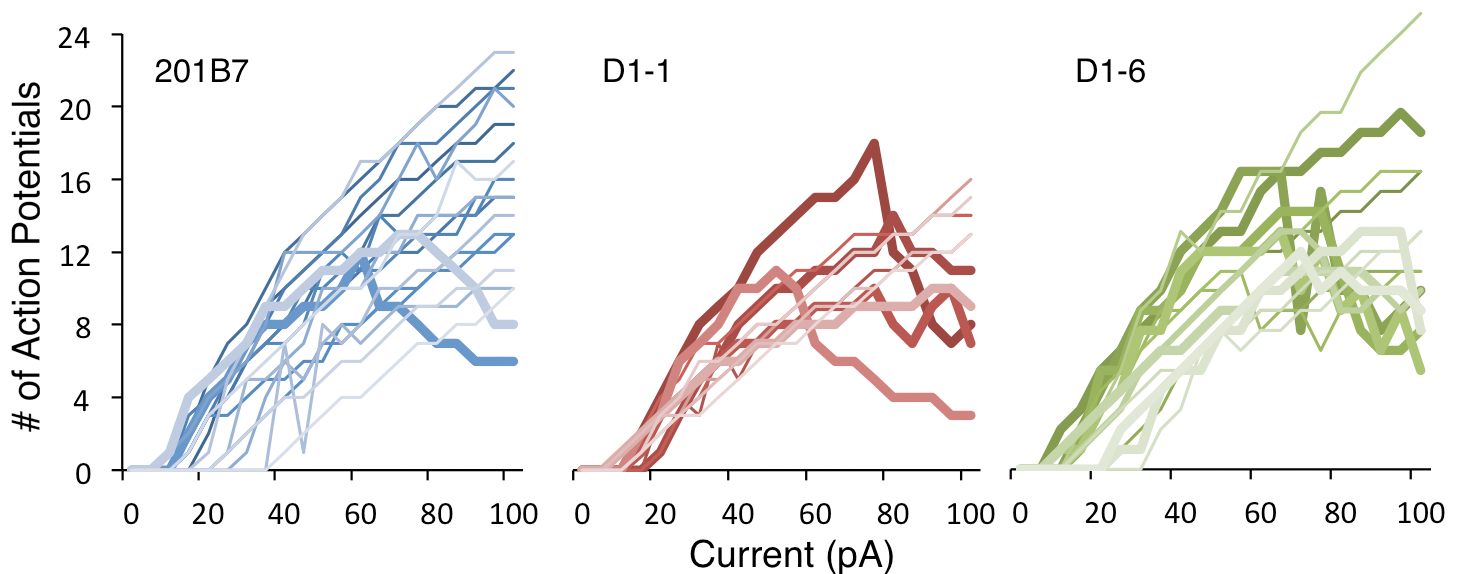

Supplement: Additional file 13 — Individual input–output relationship plots for control and Dravet-derived neurons. Experimental setup and plotting as in Additional file 6. Each line plot represents one cell. Current injections of <100 pA frequently maxed out the number of action potentials triggered in patient neurons (D1-1 and D1-6), but only rarely in the control neurons (201B7). [file 1756-6606-6-19-S13.tiff]
