# Supplementary material for: A human Dravet syndrome model from patient induced pluripotent stem cells
Source: Mol Brain. 2013 May 2;6:19. doi: 10.1186/1756-6606-6-19 (PMC3655893; doi:10.1186/1756-6606-6-19)
Supplement: Additional file 2 — Real-time PCR efficiency, Nav genes. [file 1756-6606-6-19-S2.pdf]

Real-time PCR efficiency, Na_v genes

Gene	Slope of standard curve	PCR efficiency
SCN1A	-3.334	1.99
SCN2A	-3.357	1.99
SCN3A	-3.255	2.03
SCN8A	-3.32	2.00

PCR efficiency (E) was calculated from $E = 10^{(-1/k)}$, where k is the slope of the standard curve. The respective primer sequences are compiled in Table 1.

Table 2: Primer details for real-time PCR

Target	Forward	Reverse
β-actin	GATCAAGATCATTGCTCCTCCT	GGGTGTAACGCAACTAAGTCA
Sox2 (tg[*])	ACGGCCATTAACGGCACACTG	CCCTTTTTCTGGAGACTAAATAAA
Klf4 (tg)	CACCTCGCCTTACACATGAAGAG	
Oct3/4 (tg)	TCTGGGCTCTCCCATGCATTCAAAC	
c-Myc (tg)	CTTGAACAGCTACGGAACTCTTG	
SCN1A	AACAGAATCAGGCCACCTTG	CACTGGGCTCTCTGGAATG
SCN2A	GCTACACGAGCTTTGACACC	CCCAAGAAAATGACCAGCAC
SCN3A	ATGGTGTGGTTTCCTTGGTG	TGACTTCCGTTTCTGTGGTG
SCN8A	GGACCCATGGAACTGGTTAG	ACCCTGAAAGTGCGTAGAGC
Nkx2.1	AGCACACGACTCCGTTCTCA	CCCTCCATGCCCACTTTCTT

All sequences are displayed in 5’-to-3’ direction. [*]tg, transgene
